# Supplementary material for: Multifaceted effects of variable biotic interactions on population stability in complex interaction webs
Source: Commun Biol. 2024 Oct 22;7:1309. doi: 10.1038/s42003-024-06948-2 (PMC11496648; doi:10.1038/s42003-024-06948-2)
Supplement: Supplementary file 2 — Supplementary Information [file 42003_2024_6948_MOESM2_ESM.pdf]

Supplementary materials for "Multifaceted effects of variable biotic interactions on population stability in complex interaction webs"

Authors and affiliations

Koya Hashimoto[1,2,3,*,†], Daisuke Hayasaka[1,†], Yuji Eguchi[4], Yugo Seko[2,4], Ji Cai[5], Kenta Suzuki[6,7], Koichi Goka[2], and Taku Kadoya[2,†]

1: Faculty of Agriculture, Kindai University, Nakamachi 3327-204, Nara, Nara 631-8505, Japan.

2: National Institute for Environmental Studies (NIES), Onogawa 16-2, Tsukuba, Ibaraki 305-8506, Japan.

3: Faculty of Agriculture and Life Science, Hirosaki University, 3 Bunkyo-cho, Hirosaki, Aomori 036-8561, Japan.

4: Graduate School of Agriculture, Kindai University, Nakamachi 3327-204, Nara, Nara 631-8505, Japan.

5: Center for Ecological Research, Kyoto University, Hirano 2-509-3, Otsu, Shiga 520-2113, Japan.

6: BioResource Research Center, RIKEN, Takanodai 3-1-1, Tsukuba, Ibaraki 305-0074, Japan.

7: Institute for Multidisciplinary Sciences, Yokohama National University, Tokiwadai 9-5, Hodogaya, Yokohama, Kanagawa 240-8501, Japan.

[*]For correspondence (atrophaneura4@gmail.com (K. Hashimoto))

[†]These authors contributed equally to this work.

Supplementary Note 1. Biological interpretations of the observed interaction networks.

Cascading positive effects from phytoplankton to crustacean zooplankton

Phytoplankton may be food resources for rotifers, and rotifers may be foraged by larger crustacean zooplankton. Negative effects of higher trophic levels were observed only for rotifers to phytoplankton, suggesting that bottom-up effects were more prevalent in these mesocosms. Indeed, paddy systems are more likely than other freshwater systems to be donor-controlled[1].

Phytoplankton had negative effects on detritivores and herbivores

Phytoplankton may negatively affect freshwater biodiversity by modifying water conditions, leading to conditions such as eutrophication and increased turbidity[2]. The observed negative effects on these macroinvertebrates may reflect such negative effects on water conditions.

Macrophytes had a positive effect on neustonic predators

Macrophytes gather small arthropods, such as aphids and other hemipterans, above the water surface. These small arthropods occasionally drop onto the water surface and may serve as food resources for neustonic predators (water striders).

Bidirectional effects between rotifers and phytophilous predators

Bidirectional effects between rotifers and phytophilous predators strongly suggest a prey–predator interaction. Phytophilous predators, i.e., damselfly and dragonfly larvae, are known to prey upon rotifers, especially in their earlier stages[3]. Thus, the negative effects of phytophilous predators on rotifers are reasonable. In addition, the positive effects of phytophilous predators on phytoplankton suggest top-down trophic cascades via the predation of rotifers by phytophilous predators. On the other hand, because

phytophilous predators were able to escape from the mesocosms after their development was complete, the observed negative effects of rotifers on phytophilous predators (in I, H, and I+H) do not necessarily mean that rotifers have negative effects on the survival, development, and fitness of phytophilous predators.

Negative effects of benthic predators on molluscs

Benthic predators (dragonfly larvae) may prey upon young molluscs (snails and clams). This may be reflected by the apparent positive effects of the insecticide (fipronil) on mollusc density because benthic predators were strongly negatively affected by insecticide application, suggesting density-mediated indirect effects of the insecticide on molluscs by decreasing predation by benthic predators on molluscs.

Supplementary Note 2. Rationale for the data combining method in empirical dynamic modelling.

Our method generally followed the guidelines suggested by Munch et al.[4] ("3.1 Short time series: Leveraging replicates" PP. 735-736), who mentioned that multiple time series showing similar dynamics can be combined to obtain better global predictions than would be obtainable using just a single short time series[4]. This suggestion would support our approach of combining short time series from different treatments if we can consider that the dynamics of the time series are sufficiently similar among different treatments. Furthermore, relevant approaches that combine similar but ecologically different (i.e., different species or experimental treatments) time series together for a single state-space reconstruction have been used by several previous studies[5,6] and introduced by the tutorial of the rEDM package (https://ha0ye.github.io/rEDM/articles/rEDM.html#community-productivity-and-invasibility).

In light of the above suggestion, we do not need to assume that a reconstructed attractor is exactly identical among different treatments (Attached Fig. 1a). Rather, our method can be considered to make a prediction by using a *set* of reconstructed attractors that are assumed to share a common dynamical function (and thus share similar shapes) but may be characterized by different parameter values (and thus, their relative size and/or space they occupy would be different) (Attached Fig. 1b). Assembling these different but similar attractors results in a single "thick" reconstructed attractor, the thickness of which corresponds to potentially different interaction properties and underlying parameter values of different treatments (Attached Fig. 1b).

The strength of using such a set of attractors is that the data shortage in a single reconstructed attractor from each treatment (Attached Fig. 1c) can be compensated for by other adjacent attractors, as long as the shapes of the attractors are sufficiently similar (attached Fig. 1d). Although it may sound more natural to construct treatment-

specific attractors separately, in our case, coping with information shortages would be a more critical issue. In fact, poor RMSE (approx. 2-6) values were shown by the S-map methods when treatment-specific attractors were used separately (Attached Fig. 2). On the other hand, the RMSE was substantially decreased when all the data were combined (approx. < 0.1-1) (Attached Fig. 2). This suggests that the shapes of attractors were sufficiently similar among different treatments; thus, we believe that the data combination successfully compensated for the data shortage of individual treatments.

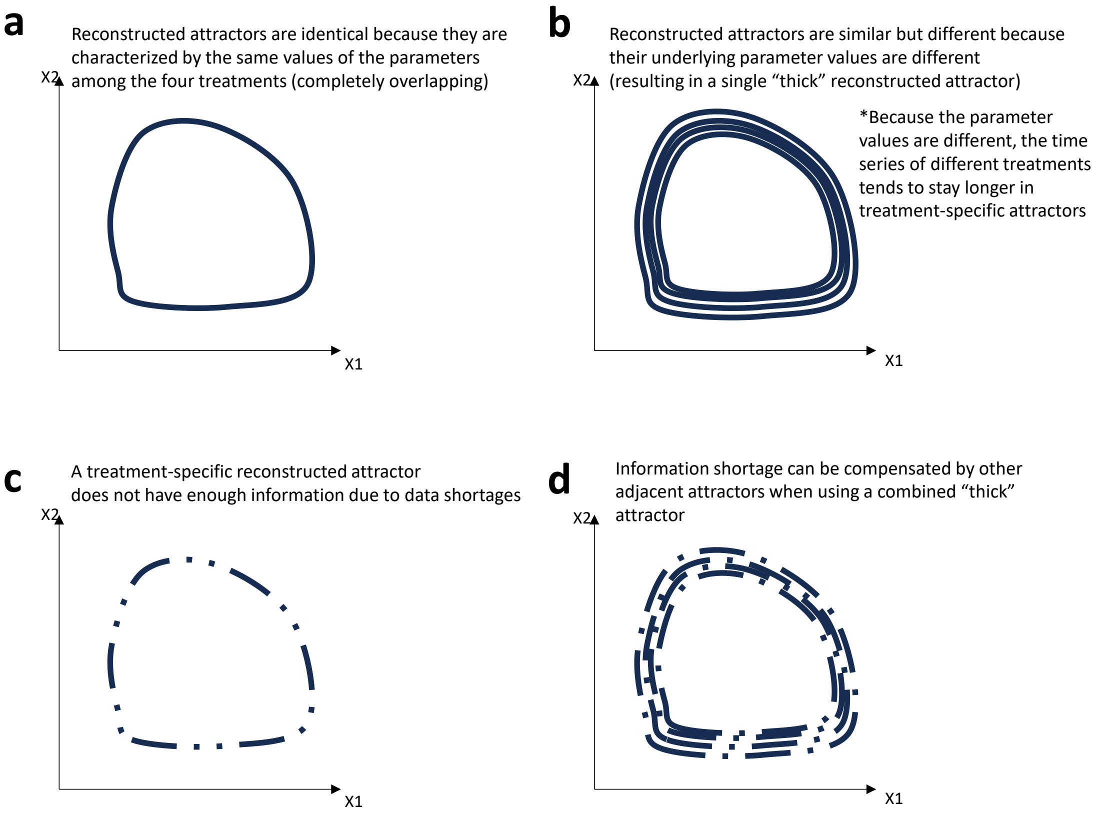

Attached Fig. 1. Four situations of attractor reconstruction when analysing different treatments together. Descriptions are shown in each panel.

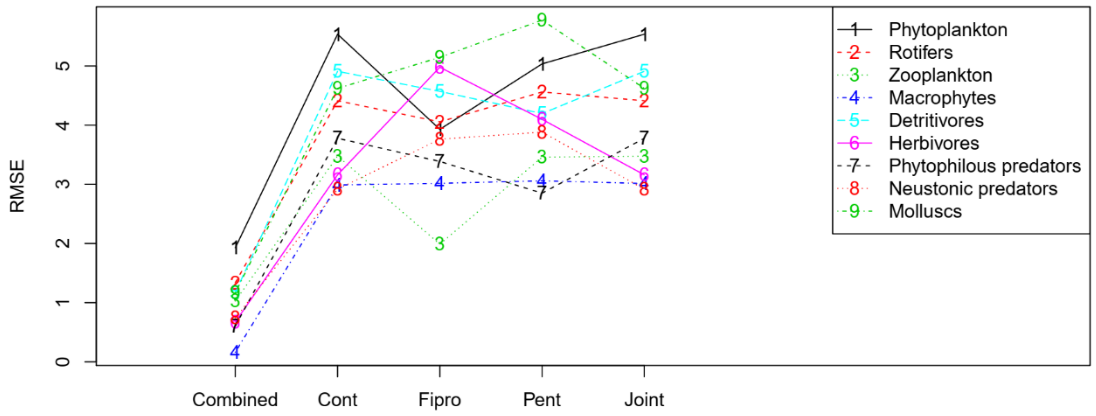

Attached Fig. 2. Forecasting skills of S-map models using combined and single reconstructed attractors. The RMSE values are shown. Cont: controls, Fipro: insecticide only, Pent: herbicide only, Joint: both insecticide and herbicide.

Supplementary Table 1. Representative taxa of the paddy community members.

Community members	Representative taxa
Eukaryotic phytoplankton	*Cryptomonas* spp. (algae)
	Euglena spp. (phyotosynthetic flagellates)
	Cosmarium spp. (algae)
	Pennate diatoms (diatoms)
Rotifers	Brachionidae
	Lecanidae
	Synchaetidae
Crustacean zooplankton	Chydoridae (cradocerans)
	Ostracoda
	Copepoda
Macrophytes	*Monochoria vaginalis* (pondweed)
	Schoenoplectiella hotarui (sedge)
	Azolla sp. (water fern)
Detritivorous insects	Chironomidae (nonbiting midges)
	Aedes spp. (mosquitos)
	Anopheles spp. (mosquitos)
Herbivorous insects	Corixidae spp. (water boatmen)
	Curculionoidea spp. (weevils)
	Pyraloidea spp. (snout moths)
Phytophilous predatory insects	*Indolestes peregrinus* (damselfly)
	Cercion calamorum (damselfly)
	Anax parthenope (dragonfly)
Benthic predatory insects	*Crocothemis servilia* (dragonfly)
	Orthetrum albistylum (dragonfly)
Neustonic predatory insects	*Microvelia douglasi* (small water strider)
	Gerris gracilicornis (water strider)
Molluscs	*Physa acuta* (snail)
	Bivalvia spp. (freshwater clams)

Supplementary Table 2. The effects of pesticide treatments on the standardized density of each community member based on linear mixed models. For fixed effects (treatment, week, and treatment × week interaction), the results of Type III likelihood ratio tests are shown. For random effects (AR1 temporal autocorrelation, tank identity, year, and residuals), the estimated autocorrelation coefficient ρ or variance σ^2 is shown. Bold indicates statistical significance.

	Treatment			Week			Treatment × Week		
	LR-χ^2	df	*P*	LR-χ^2	df	*P*	LR-χ^2	df	*P*
Eukaryotic phytoplankton	8.24	3	**0.04**	34.64	9	**< 0.001**	36.97	27	0.10
Rotifers	1.86	3	0.6	24.54	9	**< 0.01**	36.94	27	0.10
Crustacean zooplankton	2.89	3	0.4	76.86	9	**< 0.001**	29.81	27	0.3
Macrophytes	14.39	3	**< 0.01**	172.70	9	**< 0.001**	31.37	27	0.3
Detritivorous insects	5.93	3	0.1	44.58	9	**< 0.001**	23.22	27	0.7
Herbivorous insects	13.03	3	**< 0.01**	23.84	9	**< 0.01**	65.23	27	**< 0.001**
Phytophilous predatory insects	14.54	3	**< 0.01**	23.73	9	**< 0.01**	27.66	27	0.4
Benthic predatory insects	22.43	3	**< 0.001**	24.68	9	**< 0.01**	35.92	27	0.1
Neustonic predatory insects	4.86	3	0.2	46.82	9	**< 0.001**	30.17	27	0.3
Molluscs	11.49	3	**< 0.01**	11.62	9	0.2	26.78	27	0.5

	AR1 ρ	σ^2_Tank	σ^2_Year	σ^2_residual
Eukaryotic phytoplankton	0.58	8.0×10^{-10}	2.1×10^{-2}	0.64
Rotifers	0.30	0.021	0.40	0.65
Crustacean zooplankton	0.47	5.1×10^{-6}	2.2×10^{-6}	0.77
Macrophytes	0.90	2.0×10^{-4}	0.10	0.44
Detritivorous insects	0.27	0.059	0.029	0.80
Herbivorous insects	0.16	1.3×10^{-5}	5.0×10^{-2}	0.74
Phytophilous predatory insects	0.43	4.9×10^{-8}	0.11	0.71
Benthic predatory insects	0.21	0.038	4.7×10^{-2}	0.46
Neustonic predatory insects	0.40	0.075	0.017	0.85
Molluscs	0.42	0.055	0.51	0.48

Supplementary Table 3. Summary of convergent cross-mapping. The rows and columns represent recipient and donor community members, respectively. $\Delta\rho$: the differences in cross-map skills (ρ) at the minimum and the maximum library lengths. P: estimated P values based on the surrogate time series analysis. Optimal lag: the time lag between the time series of donor and recipient organisms that showed the best cross-map skills. The bold numbers indicate the causes that met the above three criteria and thus were considered in this study.

	Donor organisms									
Recipient organisms	Ph	Ro	Zo	Ma	De	He	Pp	Bp	Np	Mo
Phytoplankton (Ph)	-	**$\Delta\rho = 0.28$<br>$P < 0.001$<br>Optimal lag = –1**	$\Delta\rho = 0.06$	$\Delta\rho = -0.12$	**$\Delta\rho = 0.16$<br>$P = 0.07$<br>Optimal lag = –1**	$\Delta\rho = -0.04$	**$\Delta\rho = 0.13$<br>$P < 0.05$<br>Optimal lag = –2**	$\Delta\rho = 0.13$<br>$P < 0.05$<br>Optimal lag = 2	$\Delta\rho = 0.01$	$\Delta\rho = 0.07$
Rotifers (Ro)	**$\Delta\rho = 0.19$<br>$P < 0.01$<br>Optimal lag = –2**	-	$\Delta\rho = 0.22$<br>$P < 0.05$<br>Optimal lag = 1	$\Delta\rho = -0.15$	$\Delta\rho = 0.006$	**$\Delta\rho = 0.17$<br>$P < 0.01$<br>Optimal lag = 0**	**$\Delta\rho = 0.23$<br>$P < 0.01$<br>Optimal lag = –2**	$\Delta\rho = 0.22$<br>$P < 0.01$<br>Optimal lag = 2	$\Delta\rho = 0.002$	$\Delta\rho = 0.24$<br>$P < 0.01$<br>Optimal lag =2
Crustacean zooplankton (Zo)	$\Delta\rho = -0.11$	**$\Delta\rho = 0.29$<br>$P < 0.01$<br>Optimal lag = –2**	-	$\Delta\rho = 0.17$<br>$P = 0.17$	$\Delta\rho = 0.08$	$\Delta\rho = 0.25$<br>$P < 0.01$<br>Optimal lag = 2	$\Delta\rho = 0.22$<br>$P < 0.05$<br>Optimal lag = 2	$\Delta\rho = 0.19$<br>$P = 0.06$<br>Optimal lag = 2	$\Delta\rho = 0.24$<br>$P < 0.05$<br>Optimal lag = 2	$\Delta\rho = -0.05$
Macrophytes (Ma)	$\Delta\rho = 0.35$<br>$P < 0.001$<br>Optimal lag = 1	**$\Delta\rho = 0.28$<br>$P < 0.01$<br>Optimal lag = –2**	$\Delta\rho = 0.25$<br>$P < 0.05$<br>Optimal lag = 2	-	$\Delta\rho = 0.21$<br>$P < 0.001$<br>Optimal lag = 2	**$\Delta\rho = 0.27$<br>$P < 0.001$<br>Optimal lag = –1**	**$\Delta\rho = 0.15$<br>$P < 0.05$<br>Optimal lag = –1**	$\Delta\rho = 0.31$<br>$P < 0.001$<br>Optimal lag = 2	$\Delta\rho = 0.21$<br>$P < 0.001$<br>Optimal lag = 2	$\Delta\rho = 0.12$<br>$P < 0.05$<br>Optimal lag = 1
Detritivores (De)	**$\Delta\rho = 0.22$<br>$P < 0.01$<br>Optimal lag = 0**	**$\Delta\rho = 0.10$<br>$P = 0.09$<br>Optimal lag = –2**	$\Delta\rho = 0.18$<br>$P = 0.18$	**$\Delta\rho = 0.14$<br>$P < 0.01$<br>Optimal lag = –2**	-	$\Delta\rho = 0.13$<br>$P = 0.06$<br>Optimal lag = 2	$\Delta\rho = -0.12$	$\Delta\rho = 0.08$	$\Delta\rho = 0.07$	$\Delta\rho = 0.01$
Herbivores (He)	**$\Delta\rho = 0.14$<br>$P < 0.05$<br>Optimal lag = 0**	$\Delta\rho = 0.03$	$\Delta\rho = 0.08$	**$\Delta\rho = 0.20$<br>$P < 0.01$<br>Optimal lag = –2**	**$\Delta\rho = 0.21$<br>$P < 0.01$<br>Optimal lag = –2**	-	$\Delta\rho = 0.16$<br>$P < 0.01$<br>Optimal lag = 2	$\Delta\rho = 0.33$<br>$P < 0.001$<br>Optimal lag = 2	$\Delta\rho = 0.04$	$\Delta\rho = 0.16$<br>$P = 0.13$
Phytophilous predators (Pp)	$\Delta\rho = -0.01$	**$\Delta\rho = 0.21$<br>$P < 0.05$<br>Optimal lag = –2**	$\Delta\rho = 0.17$<br>$P = 0.18$	$\Delta\rho = -0.08$	$\Delta\rho = 0.03$	$\Delta\rho = 0.01$	-	$\Delta\rho = 0.05$	$\Delta\rho = 0.03$	$\Delta\rho = 0.02$
Benthic predators (Bp)	$\Delta\rho = 0.01$	$\Delta\rho = 0.03$	$\Delta\rho = 0.01$	$\Delta\rho = 0.003$	$\Delta\rho = -0.02$	$\Delta\rho = 0.12$<br>$P = 0.13$	$\Delta\rho = -0.003$	-	$\Delta\rho = -0.13$	$\Delta\rho = 0.05$
Neustonic predators (Np)	$\Delta\rho = 0.095$	$\Delta\rho = 0.12$<br>$P = 0.10$	$\Delta\rho = 0.11$<br>$P = 0.30$	**$\Delta\rho = 0.16$<br>$P < 0.001$<br>Optimal lag = –2**	$\Delta\rho = 0.03$	$\Delta\rho = 0.06$	$\Delta\rho = 0.06$	$\Delta\rho = 0.14$<br>$P = 0.09$<br>Optimal lag = 1	-	$\Delta\rho = -0.04$
Molluscs (Mo)	$\Delta\rho = 0.11$<br>$P < 0.001$<br>Optimal lag = 1	**$\Delta\rho = 0.18$<br>$P < 0.001$<br>Optimal lag = –1**	**$\Delta\rho = 0.11$<br>$P = 0.09$<br>Optimal lag = –2**	$\Delta\rho = 0.10$<br>$P = 0.11$	$\Delta\rho = 0.11$<br>$P = 0.19$	**$\Delta\rho = 0.13$<br>$P < 0.05$<br>Optimal lag = 0**	$\Delta\rho = 0.17$<br>$P < 0.05$<br>Optimal lag = 1	**$\Delta\rho = 0.17$<br>$P < 0.001$<br>Optimal lag = –2**	**$\Delta\rho = 0.27$<br>$P < 0.01$<br>Optimal lag = –1**	-

Supplementary Table 4. Embedding dimension and nonlinearity of each single-variable and multivariate embedding. *E*: Optimal embedding dimension determined by simplex projection (for multivariate embedding, the dimension actually used was reported). *θ*: best nonlinearity parameters determined by the S-map method. *λ*: the L2 penalization parameter to avoid overfitting. Recipients and donors: receivers and initiators of interaction effects determined by convergent cross-mapping, respectively.

Embedding				
Single variate embedding†		*E*	*θ*	
Eukaryotic phytoplankton		5	0.75	
Rotifers		6	1.5	
Crustacean zooplankton		6	1	
Macrophytes		5	0.3	
Detritivorous insects		4	2	
Herbivorous insects		6	1	
Phytophilous predatory insects		5	0.75	
Benthic predatory insects		2	3	
Neustonic predatory insects		5	0.03	
Molluscs		4	0.75	
Multivariate embedding				
Recipients	Donors	*E*	*θ*	*λ*
Eukaryotic phytoplankton	Rotifers, Detritivorous insects, Phytophilous predatory insects	5	2	0.01
Rotifers	Eukaryotic phytoplankton, Herbivorous insects, Phytophilous predatory insects	6	1	0.1
Crustacean zooplankton	Rotifers	6	0	0.1
Macrophytes	Rotifers, Herbivorous insects, Phytophilous predatory insects	5	6	0.1
Detritivorous insects	Eukaryotic phytoplankton, Rotifers, Macrophytes	4	2.5	0
Herbivorous insects	Eukaryotic phytoplankton, Macrophytes, Detritivorous insects	6	0	0.01
Phytophilous predatory insects	Rotifers	5	8	2
Neustonic predatory insects	Macrophytes	5	0	0.01
Molluscs	Rotifers, Crustacean zooplankton, Herbivorous insects, Benthic predatory insects, Neustonic predatory insects	5	6	1

†: Ordinary S-map instead of regularized S-map was used.

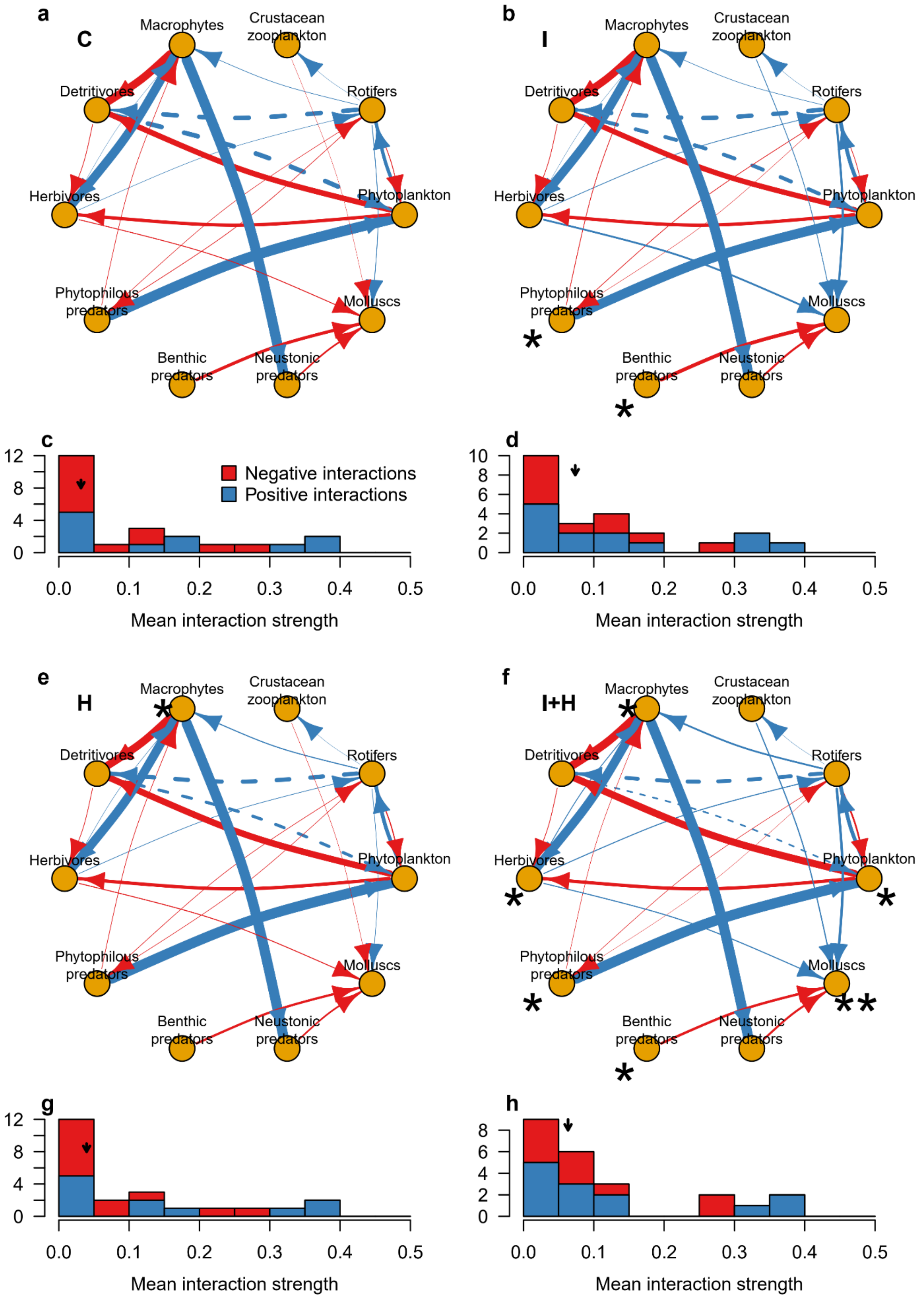
a
C
b
I
e
H
f
I+H
Macrophytes
Crustacean zooplankton
Detritivores
Rotifers
Herbivores
Phytoplankton
Phytophilous predators
Molluscs
Benthic predators
Neustonic predators
c
d
g
h
Negative interactions
Positive interactions
Mean interaction strength
0.0
0.1
0.2
0.3
0.4
0.5
0
4
8
12
0
2
4
6
8
10

Supplementary Fig. 1. Reconstructed interaction networks of each pesticide treatment by EDM analyses. C: control, I: insecticide (fipronil) treatment, H: herbicide (pentoxazone) treatment, and I+H: insecticide + herbicide mixture treatment. In **a**, **b**, **e**, and **f**, the red and blue arrows indicate negative and positive interactions, respectively. Their thickness is proportional to the *per capita* interaction effects represented by the absolute values of the S-map coefficient averaged over all the experimental periods and replicates. Solid arrows: $P < 0.05$ and dashed arrows: $0.05 < P < 0.1$. One asterisk in **b**, **e** and **f** indicates that the indicated member was significantly decreased by the pesticide treatment relative to the controls, whereas two asterisks in **f** indicate that the indicated member was significantly increased by the pesticide treatment relative to the controls. In **c**, **d**, **g**, and **h**, the distributions of the absolute values of the S-map coefficient averaged over all the experimental periods and replicates are shown by histograms. The vertical arrows indicate the median values.

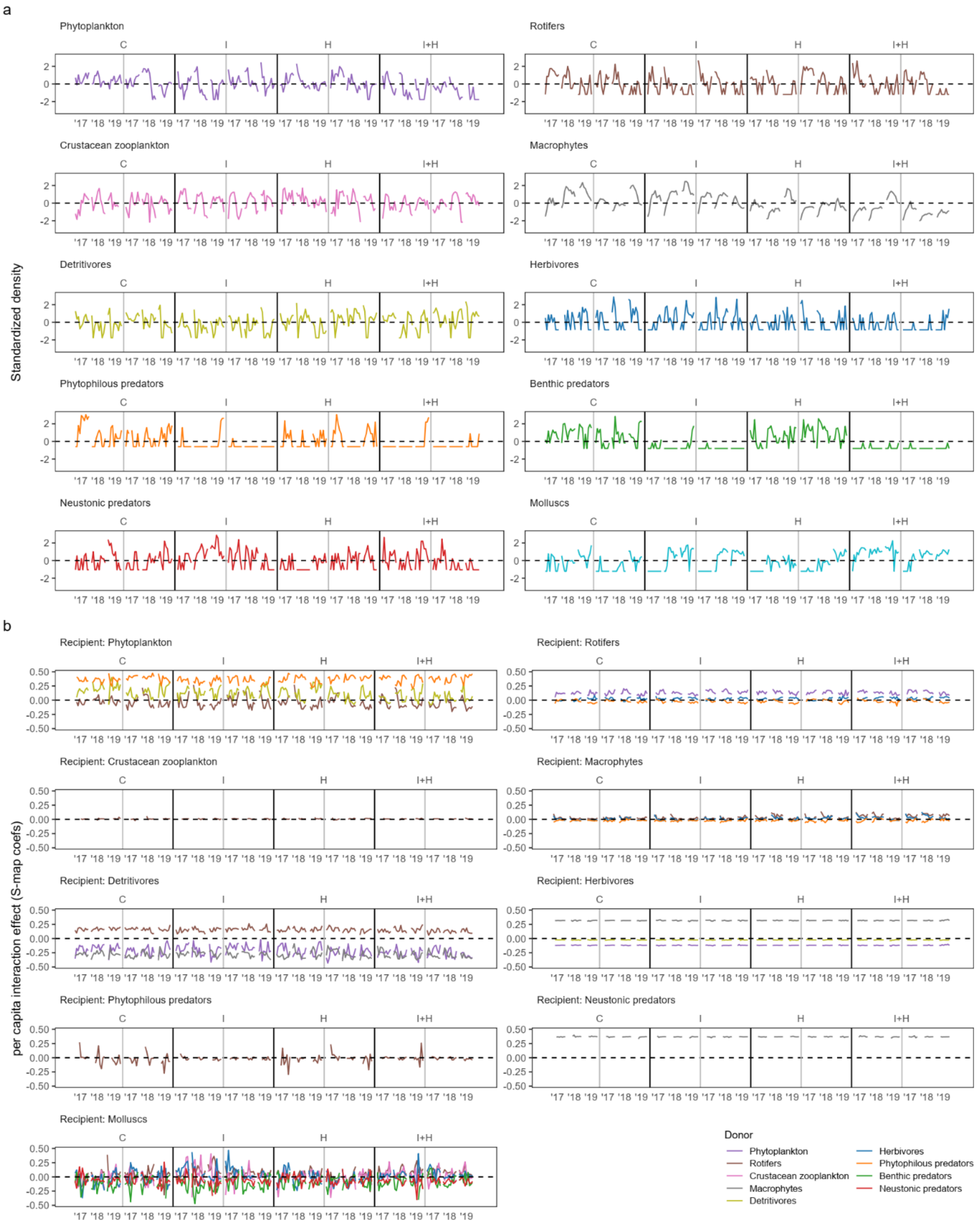

Supplementary Fig. 2. Time series of a) standardized densities of community members in experimental paddies and b) *per capita* interaction effect (S-map coefficients) of all the treatments and replicates. The panels are split by interaction recipients.

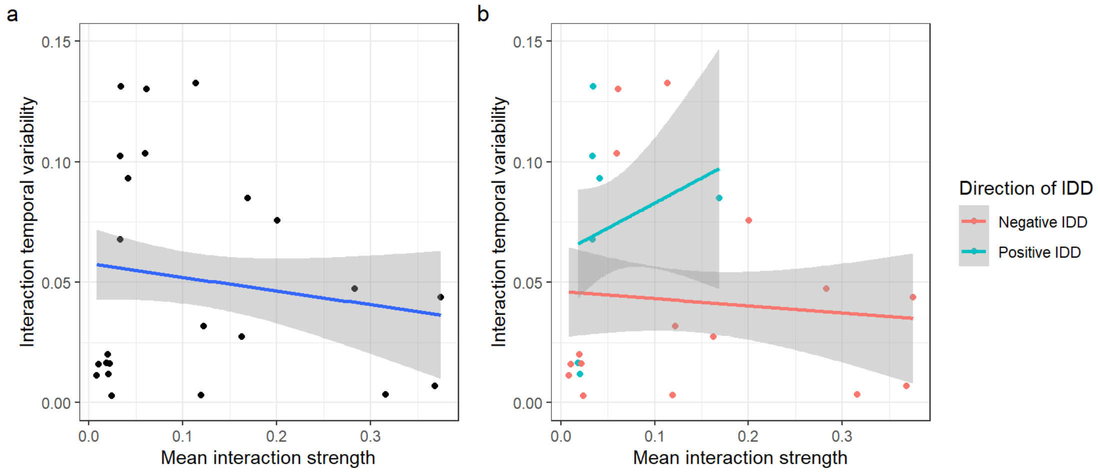

Supplementary Fig. 3. Correlations between mean interaction strength and interaction temporal variability. a and **b** use the same data, but the latter panel explicitly shows the direction of recipient density-dependence in *per capita* interaction effect (interaction density-dependence; IDD). Although the correlation was not statistically significant ($P = 0.2$), some of the weaker interactions were temporally variable, whereas the stronger interactions were relatively temporally stable. This pattern was mainly driven by interactions with negative IDD.

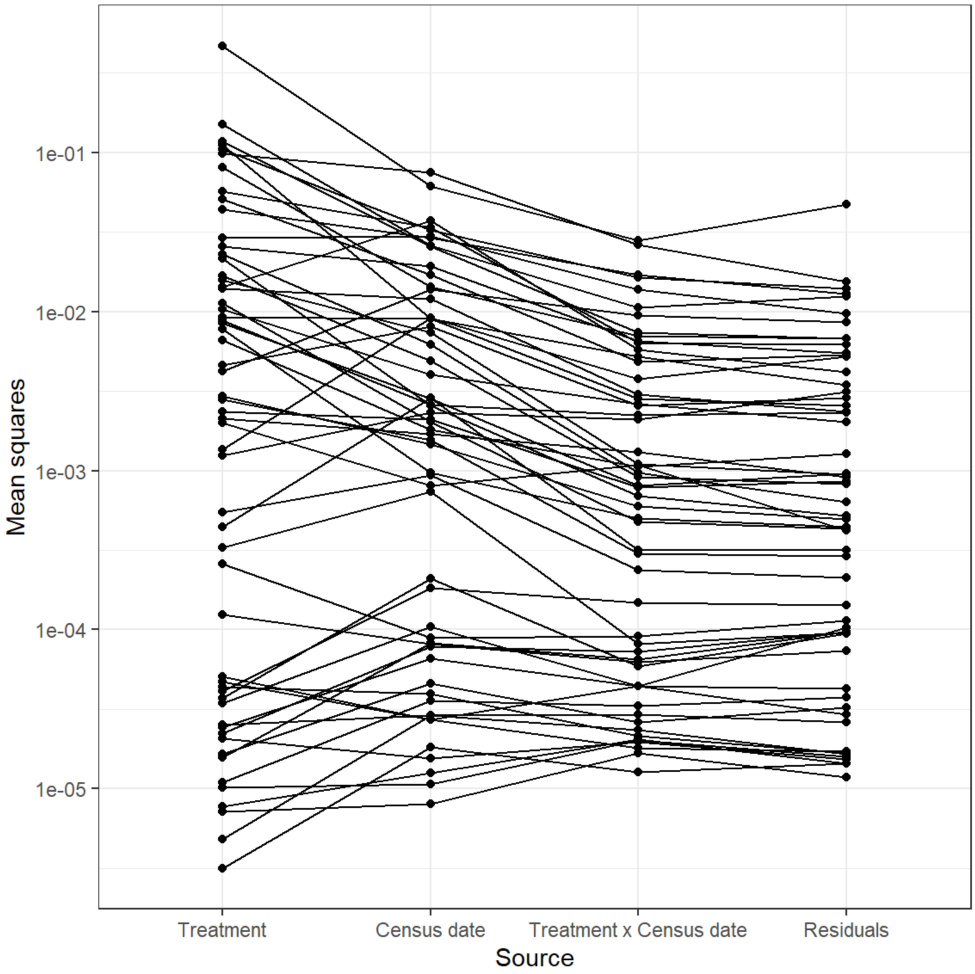

Supplementary Fig. 4. Contribution of the source of variation to the values of S-map coefficients evaluated by mean squares. Each solid line represents one ANOVA model examining the effects of treatment, census week, their interaction and residuals on the value of an S-map coefficient. Of the 57 S-map coefficients, for 41 coefficients, the contribution of treatment was greater than that of residuals, and for 48 coefficients, the contribution of census date was greater than that of residuals.

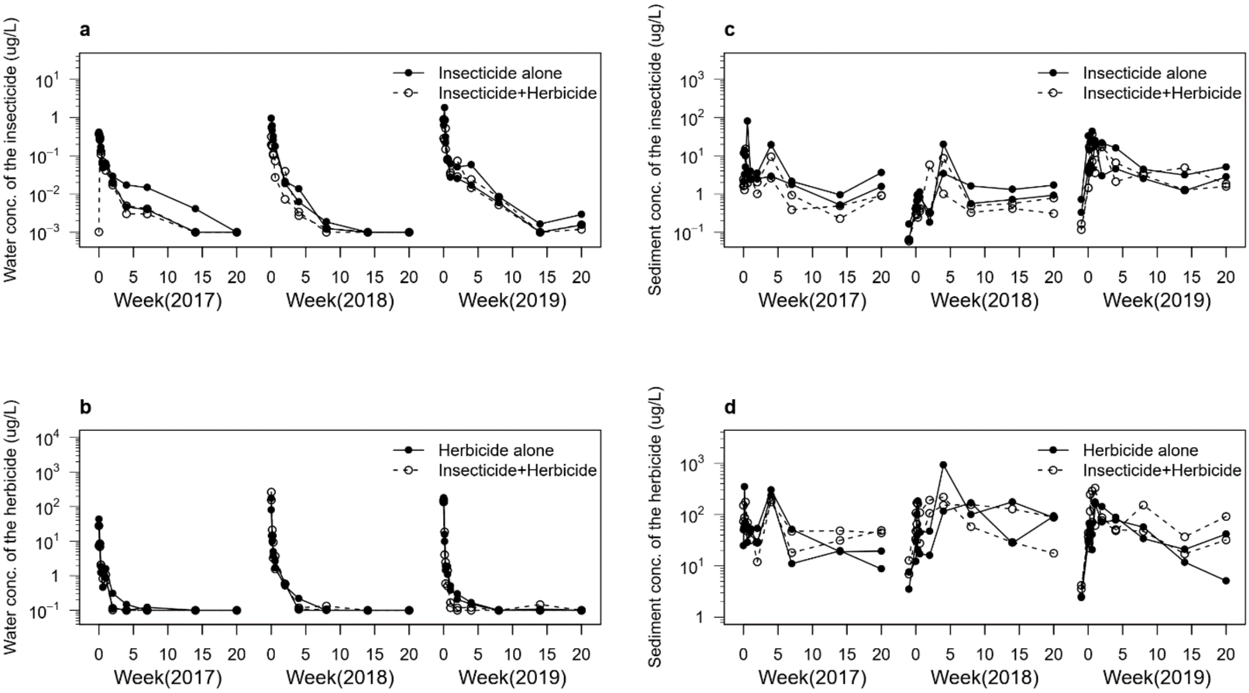

Supplementary Fig. 5. Temporal dynamics of pesticides in the experimental mesocosms. **a**, **b**, Concentration of **a**) the insecticide (fipronil) and **b**) the herbicide (pentoxazone) in water. **c**, **d**, Concentration of **c**) the insecticide and **d**) the herbicide in sediment. The data below the limits of detection were substituted with limit values to facilitate visual interpretation.

Note: We collected water (50 mL) from 20 random sampling locations (2.5 mL per spot) in each pesticide-treated mesocosm. We also collected surface sediment (100 g, 2–3 cm depth) from 10 random sampling locations (10 g per spot) in each mesocosm. To avoid photolysis and degradation of both pesticides, we placed the collected samples in amber bottles sealed with aluminium foil and stored them in a refrigerator (5 °C) until analysis. Analyses of pentoxazone and fipronil in water and sediment were carried out at the certified analytical laboratories of HEISEIRIKEN Co., Ltd. (Utsunomiya, Tochigi Prefecture, Japan) using LC–MS/MS analysis.

Supplementary references

1. Okuda, N. How to assess ecosystem structure and functioning of paddy fields using stable isotopes. *Japanese J. Ecol.* **62**, 207–216 (2012). (in Japanese)
2. Scheffer, M., Carpenter, S., Foley, J. A., Folke, C. & Walker, B. Catastrophic shifts in ecosystems. *Nature* **413**, 591–596 (2001).
3. Sugita, N., Agemori, H. & Goka, K. Acute toxicity of neonicotinoids and some insecticides to first instar nymphs of a non-target damselfly, Ischnura senegalensis (Odonata: Coenagrionidae), in Japanese paddy fields. *Appl. Entomol. Zool.* **53**, 519–524 (2018).
4. Munch, S. B., Rogers, T. L. & Sugihara, G. Recent developments in empirical dynamic modelling. *Methods Ecol. Evol.* **14**, 732–745 (2022).
5. Hsieh, C., Anderson, C. & Sugihara, G. Extending nonlinear analysis to short ecological time series. *Am. Nat.* **171**, 71–80 (2008).
6. Virtanen, R., Clark, A. T., den Herder, M. & Roininen, H. Dynamic effects of insect herbivory and climate on tundra shrub growth: Roles of browsing and ramet age. *J. Ecol.* **109**, 1250–1262 (2021).
