# Supplementary material for: Multifaceted effects of variable biotic interactions on population stability in complex interaction webs
Source: Commun Biol. 2024 Oct 22;7:1309. doi: 10.1038/s42003-024-06948-2 (PMC11496648; doi:10.1038/s42003-024-06948-2)
Supplement: Supplementary file 3 — Reporting Summary [file 42003_2024_6948_MOESM3_ESM.pdf]

nature portfolio

Corresponding author(s): Koya Hashimoto

Last updated by author(s): Sep 11, 2024

Reporting Summary

Nature Portfolio wishes to improve the reproducibility of the work that we publish. This form provides structure for consistency and transparency in reporting. For further information on Nature Portfolio policies, see our Editorial Policies and the Editorial Policy Checklist.

Statistics

For all statistical analyses, confirm that the following items are present in the figure legend, table legend, main text, or Methods section.

n/a	Confirmed	
☐	☒	The exact sample size (*n*) for each experimental group/condition, given as a discrete number and unit of measurement
☐	☒	A statement on whether measurements were taken from distinct samples or whether the same sample was measured repeatedly
☐	☒	The statistical test(s) used AND whether they are one- or two-sided<br>*Only common tests should be described solely by name; describe more complex techniques in the Methods section.*
☐	☒	A description of all covariates tested
☐	☒	A description of any assumptions or corrections, such as tests of normality and adjustment for multiple comparisons
☐	☒	A full description of the statistical parameters including central tendency (e.g. means) or other basic estimates (e.g. regression coefficient) AND variation (e.g. standard deviation) or associated estimates of uncertainty (e.g. confidence intervals)
☐	☒	For null hypothesis testing, the test statistic (e.g. F, t, r) with confidence intervals, effect sizes, degrees of freedom and P value noted<br>*Give P values as exact values whenever suitable.*
☒	☐	For Bayesian analysis, information on the choice of priors and Markov chain Monte Carlo settings
☐	☒	For hierarchical and complex designs, identification of the appropriate level for tests and full reporting of outcomes
☐	☒	Estimates of effect sizes (e.g. Cohen's *d*, Pearson's *r*), indicating how they were calculated

Our web collection on statistics for biologists contains articles on many of the points above.

Software and code

Policy information about availability of computer code

Data collection	Not applicable.
Data analysis	All data analyses were conducted using the statistical environment 'R' version 3.6.3. The EDM analyses were conducted using the package 'rEDM' version 0.7.5. The code used in this study are freely available in the Zenodo (https://doi.org/10.5281/zenodo.13609190) and github (https://github.com/KoyaHashimoto/PaddyInteractionVariability) repositories.

For manuscripts utilizing custom algorithms or software that are central to the research but not yet described in published literature, software must be made available to editors and reviewers. We strongly encourage code deposition in a community repository (e.g. GitHub). See the Nature Portfolio guidelines for submitting code & software for further information.

Data

Policy information about availability of data

All manuscripts must include a data availability statement. This statement should provide the following information, where applicable:

- Accession codes, unique identifiers, or web links for publicly available datasets
- A description of any restrictions on data availability
- For clinical datasets or third party data, please ensure that the statement adheres to our policy

The data presented in this study are freely available in the Zenodo (https://doi.org/10.5281/zenodo.13609190) and github (https://github.com/KoyaHashimoto/PaddyInteractionVariability) repositories.

Human research participants

Policy information about studies involving human research participants and Sex and Gender in Research.

Reporting on sex and gender	Not applicable.
Population characteristics	Not applicable.
Recruitment	Not applicable.
Ethics oversight	Not applicable.

Note that full information on the approval of the study protocol must also be provided in the manuscript.

Field-specific reporting

Please select the one below that is the best fit for your research. If you are not sure, read the appropriate sections before making your selection.

☐ Life sciences ☐ Behavioural & social sciences ☒ Ecological, evolutionary & environmental sciences

For a reference copy of the document with all sections, see nature.com/documents/nr-reporting-summary-flat.pdf

Ecological, evolutionary & environmental sciences study design

All studies must disclose on these points even when the disclosure is negative.

Study description	In March of 2017, we buried eight independent, fiber-reinforced plastic (FRP) tanks (280 cm length × 120 cm width × 40 cm depth) on the ground in the campus facilities of Kindai University (Nara prefecture, Japan). Then, we spread sediments of uncontaminated areas near the study site on the bottom of each tank (approximately 30 cm depth). Each tank was randomly assigned to one of the four treatments, i.e., C: controls, I: insecticide alone, H: herbicide alone, and I+H: mixture of insecticide and herbicide. During 2017–2019, we repeated the following procedure. At mid-April, the mesocosms were flooded with dechlorinated water to a depth of approximately 5 cm. At the end of May to early June, we transplanted insecticide-treated and control rice seedlings (Hino-hikari variety) with an array of 4 × 10 with 25 cm intervals. We applied the insecticide (fipronil) and the herbicide (pentoxazone) in the same way as recommended for commercial rice fields. We treated nursery boxes of rice seedlings with Prince ® (1% granular fipronil, HOKKO Chemical Industry, Inc., Tokyo, Japan), at a rate of 50 g/box, 24-h prior to the transplanting. Immediately after transplanting of rice seedlings, we applied Sainyoshi flowable ® (8.6% pentoxazone, KAKEN Pharmaceutical Co., Ltd., Tokyo, Japan), at a rate of 1.7 mL/tank (i.e., 500 mL/10-a) to the mesocosms. The experiment was terminated on mid-October (i.e., approx. 140 days). During the experiment, we monitored the density of each community member every two weeks throughout the approx. 140 days experimental period until harvest in every experimental year.
Research sample	During the experiment, we took time-series data of the density of ten paddy community members; eukaryotic phytoplankton, rotifers, crustacean zooplankton, macrophytes, and aquatic macroinvertebrates. Aquatic macroinvertebrates were further divided into the following categories: detritivorous insects, herbivorous insects, phytophilous (clinging to macrophyte stems or leaves) predatory insects, benthic (living on bottom sediment) predatory insects, neustonic (living on surface of water) predatory insects and molluscs.
Sampling strategy	Eight mesocosms of four treatments with two replicates each were sampled 30 times per mesocosm to obtain time series of community member density. Previous ecotoxicological, mesocosm studies have also used such relatively small number of replicates, typically ranged 2-5, probably because of resource limitation.
Data collection	To monitor the density of phytoplankton, rotifers and crustacean zooplankton, we took 500 mL water samples from 10 random sampling points in each experimental paddy and counted them using an optical microscope and a Sedgewick-Rafter counting chamber. Counting was conducted by Ji Cai. Note that we used different sampling protocols for crustacean zooplankton in 2017 and 2018–2019. In 2017, we sampled water (1 L) using the same method described above but filtered the samples through a 250-µm plankton net (RIGOSHA & Co., Ltd., Tokyo, Japan). Then, the zooplankton were preserved in 4% formalin and counted using a stereoscopic microscope (SMZ1500, Nikon Instech Co., Ltd., Tokyo, Japan). Macrophytes density was monitored by setting three permanent quadrats (30 × 30 cm) in each mesocosm. Macrophytes were monitored by Koya Hashimoto. We collected aquatic macroinvertebrates by scooping a fishnet (1 mm mesh size) between the edges of each tank and rice seedlings (a permanent transect). Fishnet scooping was conducted by Yuji Eguchi.
Timing and spatial scale	The experiment was conducted from May to October (approx. 140 days) of each experimental year (2017-2019). The experimental field had an area of approx. 50 m^2.
Data exclusions	Data of preliminary sampling (conducted in -1 and +1 week from starting the experiment) were excluded to fulfill the requirement of the EDM analysis where the interval of time series must be constant.
Reproducibility	We repeated the same experimental procedure three times (i.e., three years) and confirmed that almost the same organisms assembled in the mesocosms in each year and that consistent effects of the pesticide treatments on the communities in the

	mesocosms were observed every experimental year. All of our analytical results are reproducible by running the R code in https://doi.org/10.5281/zenodo.13609190 or https://github.com/KoyaHashimoto/PaddyInteractionVariability.
Randomization	We randomly assigned each tank to one of the four treatments.
Blinding	Blinding was not relevant in this study.

Did the study involve field work? ☒ Yes ☐ No

Field work, collection and transport

Field conditions	The experiment was conducted over the growing season of Japan (May-October), with air temperatures ranging between approx. 17℃ and 31℃ (mean daily temp.). The mesocosms were easily accessible because these were in the campus facilities of Kindai University.
Location	The experiment was conducted at Kindai University, Nara prefecture, Japan (34°67′ N, 135°73′ E).
Access & import/export	Samples and specimens of assembling organisms were collected from an outdoor mesocosm experiment, not from the wild.
Disturbance	Disturbance is not relevant in this study because this was conducted in the campus facilities for any field experiments.

Reporting for specific materials, systems and methods

We require information from authors about some types of materials, experimental systems and methods used in many studies. Here, indicate whether each material, system or method listed is relevant to your study. If you are not sure if a list item applies to your research, read the appropriate section before selecting a response.

Materials & experimental systems

n/a	Involved in the study
☒	☐ Antibodies
☒	☐ Eukaryotic cell lines
☒	☐ Palaeontology and archaeology
☐	☒ Animals and other organisms
☒	☐ Clinical data
☒	☐ Dual use research of concern

Methods

n/a	Involved in the study
☒	☐ ChIP-seq
☒	☐ Flow cytometry
☒	☐ MRI-based neuroimaging

Animals and other research organisms

Policy information about studies involving animals; ARRIVE guidelines recommended for reporting animal research, and Sex and Gender in Research

Laboratory animals	This study did not involve laboratory animals.
Wild animals	Zooplankton were caught with water sample and preserved in 4% formalin or 5% acid Lugol's solution to identify and count them. Insects and molluscs were collected by scooping a fishnet (1-mm mesh size) and then were preserved in 70% ethanol to identify and count them.
Reporting on sex	Not applicable.
Field-collected samples	Zooplankton sample were disposed after counting. Most of the Insect and mollusc specimen were preserved in the laboratory of Kindai University.
Ethics oversight	No ethical approval was needed because the study was conducted on freshwater invertebrate communities.

Note that full information on the approval of the study protocol must also be provided in the manuscript.
